# Identification of a novel cDC2-committed progenitor within mouse common dendritic cell progenitor population

**DOI:** 10.1007/s13238-021-00902-2

**Published:** 2022-01-03

**Authors:** Yujie Tian, Xueheng Guo, Tao Wu, Kuangyu Fei, Li Wu

**Affiliations:** 1 Institute for Immunology, Tsinghua-Peking Center for Life Sciences, School of Medicine, Tsinghua University, Beijing, 100084 China; 2 Joint Graduate Program of Peking-Tsinghua-National Institute of Biological Sciences, School of Life Sciences, Tsinghua University, Beijing, 100084 China; 3 Beijing Key Laboratory for Immunological Research on Chronic Diseases, Beijing, 100084 China; 4 Present Address: National Education Examinations Authority, Beijing, 100084 China

**Dear Editor,**

Dendritic cells (DCs) are the most efficient professional antigen presenting cells that act as sentinels of the immune system and conduct vital functions in the initiation and regulation of innate and adaptive immunities (Wculek et al., 2020). In addition to being critical cellular components in pathogen clearance, DCs are promising targets for improved tumor immunotherapy, vaccine design, and intervention of autoimmune diseases (Nutt and Chopin, 2020). DCs can be classified as plasmacytoid DCs (pDCs) and conventional DCs (cDCs) in both mouse and human (Anderson et al., 2018; Nutt and Chopin, 2020). The cDCs comprise two developmentally and functionally distinct subsets known as CD8α^+^ (also CD24^+^/XCR1^+^/CD103^+^) cDC1 and CD11b^+^ (also SIRPα^+^) cDC2 in mouse (Anderson et al., 2018; Nutt and Chopin, 2020). cDC1 are specialized in antigen cross-presentation and activation of cytotoxic T lymphocytes that are crucial effectors of cellular immunity. While cDC2 are featured as antigen presenting cells of extracellular pathogens and promoting various helper T cell differentiation, including Th2 and Th17, therefore playing essential roles in humoral and cellular immunity (Nutt and Chopin, 2020).

Given that each DC subset performs unique and irreplaceable functions in immune responses, it underpins that DC development and differentiation are indispensable in modulating DC-centric immune responses. All DC subsets can arise from Lin^−^c-Kit^int^Flt3^+^CD11c^−^IL-7R^−^ common DC progenitors (CDPs) identified within bone marrow (BM) (Naik et al., 2007; Onai et al., 2007, 2013). CDPs can be divided into two subfractions according to the expression level of CD115. The CD115^−^ CDPs are enriched for precursors with pDC differentiation potential, while CD115^+^ CDPs preferably produce more cDCs (Naik et al., 2007; Onai et al., 2007, 2013). Recent studies suggest that CD115^+^ CDPs are heterogeneous and already exhibit the transcriptional priming of the cDC1 or cDC2 lineage, leading to separate differentiation into pre-cDC1 and pre-cDC2 (Schlitzer et al., 2015). Indeed, *Zeb2*^lo^ and *Id2*^hi^ CDPs prone to produce cDC1 (Bagadia et al., 2019). However, the existence of cDC2-primed progenitors within CDPs remains to be identified. In this study, we identified a Ly6C^+^ subset amongst CD115^+^ CDPs, representing cDC2-commited progenitors.

As expression of Ly6C served as a lineage marker for distinguishing pre-cDC2, it may also be implicated in cDC2 priming during the CDP stage (Schlitzer et al., 2015; Dress et al., 2019). We therefore examined the cell-surface expression of Ly6C on CDPs and found that Ly6C was only expressed by a fraction of CD115^+^ CDPs, but not by CD115^−^ CDPs. Ly6C segregated CDPs into three subsets, namely CD115^−^Ly6C^−^, CD115^+^Ly6C^−^, and CD115^+^Ly6C^+^ CDPs (Fig. 1A). The abundance of CD115^+^Ly6C^+^ CDPs accounted for approximately 25% of CD115^+^ CDPs and 10% of total CDPs (Fig. 1B and 1C). Furthermore, the surface protein profiles of CD115^+^Ly6C^+^ CDPs revealed their distinctions from any other CD115- or Ly6C-expressing BM progenitors, including Lin^−^c-Kit^+^Flt3^+^CD115^+^CD11b^−^Ly6C^−^ macrophage DC progenitors (MDPs), Lin^−^c-Kit^+^Flt3^−^CD115^+^CD11b^−^Ly6C^+^ common monocyte progenitors (cMoPs), and c-Kit^−^Flt3^−^CD115^+^CD11b^+^Ly6C^lo^ or Ly6C^hi^ monocytes (Figs. 1D and S1A).

**Figure 1:**
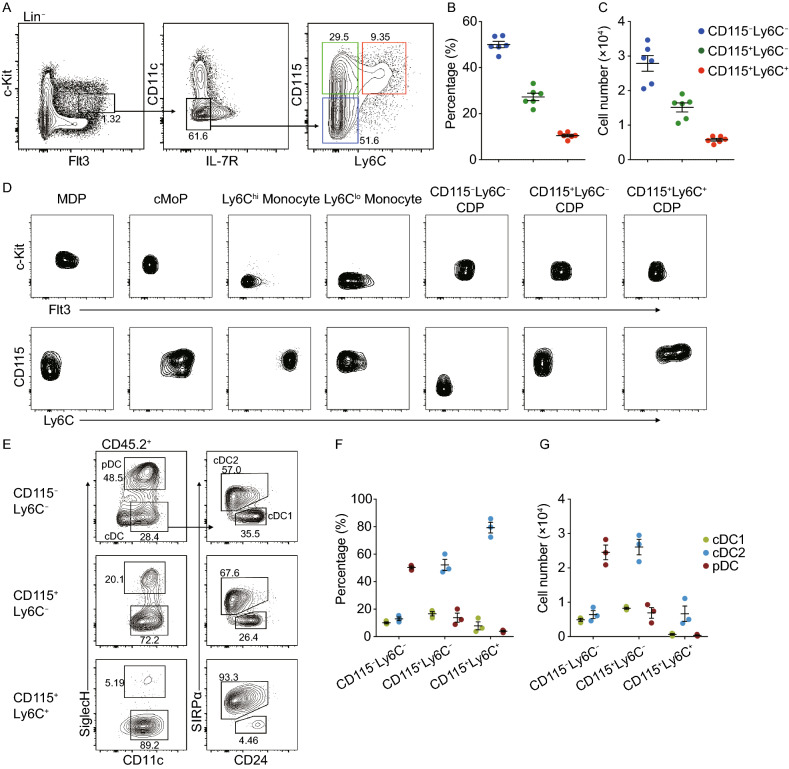
**Identification of cDC2-primed CD115**^**+**^**Ly6C**^**+**^** subset within CDPs**. (A) Flow cytometry analysis of the expression of CD115 and Ly6C on CDPs isolated from BM. Three indicated CDP subsets were identified as CD115^−^Ly6C^−^ CDPs (blue), CD115^+^Ly6C^−^ CDPs (green), and CD115^+^Ly6C^+^ CDPs (red). (B) Percentages within total CDPs and (C) cell numbers of three indicated CDP subsets defined as in (A) (*n* = 6). (D) Flow cytometry analysis of the expression of Flt3, c-Kit, CD115, and Ly6C on macrophage DC progenitors (MDPs), common monocyte progenitors (cMoPs), Ly6C^hi^ monocytes, Ly6C^lo^ monocytes, and three indicated CDP subsets. (E–G) Purified CD45.2^+^ CD115^−^Ly6C^−^, CD115^+^Ly6C^−^, and CD115^+^Ly6C^+^ CDPs were co-cultured with CD45.1^+^ total BM cells in the medium containing Flt3L (100 ng/mL), respectively. (E) Representative flow-cytometric profiles, (F) percentages, and (G) cell numbers of CD45.2^+^ DC subsets derived from indicated CDP subsets at day 3 (*n* = 3). Data in (D–G) are representative of three independent experiments. Data in (B, C, F, and G) are represented as mean ± SEM

To assess the differentiation potentials of and relationships amongst CD115^−^Ly6C^−^, CD115^+^Ly6C^−^, and CD115^+^Ly6C^+^ CDP subsets, each CDP subset was purified and cultured by *in vitro* DC differentiation system in the presence of Flt3L (Naik et al., 2005). Both CD115^−^Ly6C^−^ and CD115^+^Ly6C^−^ CDPs could give rise to CD115^+^Ly6C^+^ CDP progenies at day 1 (Fig. S1B), indicating the relative downstream position of CD115^+^Ly6C^+^ CDPs among the three CDP subsets. Furthermore, the differentiation potentials of the three CDP subsets were determined by analyzing the composition of their progenies in culture on day 3 and 6. Compared with CD115^−^Ly6C^−^ and CD115^+^Ly6C^−^ CDPs, CD115^+^Ly6C^+^ CDPs predominantly produced SIRPα^+^ cDC2 on day 3, the time to obtain maximum amounts of cDC products (Figs. 1E–G and S2A), implying their commitment toward the cDC2 lineage. Moreover, the CD115^+^Ly6C^+^ CDPs displayed lower proliferative capacity than that of the other two CDP subsets (Fig. S2A). Consequently, these results suggested that the CD115^+^Ly6C^+^ CDPs gave rise mainly to cDC2, with developmentally more mature properties compared to CD115^−^Ly6C^−^ and CD115^+^Ly6C^−^ CDPs.

To further confirm the developmental potentials of the three CDP subsets *in vivo*, we performed adoptive transfer of the three CDP subsets. At 10 days post transfer, the numbers of progenies derived from the three subsets of CDP all peaked in the recipient spleen (Fig. S2B). Consistent with the results of *in vitro* cultures, CD115^+^Ly6C^+^ CDPs generated predominantly cDC2, but little pDC and cDC1 subsets in the spleen (Fig. 2A–C). Flow cytometry analysis demonstrated that, cDC2 derived from CD115^+^Ly6C^−^ and CD115^+^Ly6C^+^ CDPs expressed similar levels of SIRPα, CD11b, ESAM, and CD4, while cDC2 derived from CD115^−^Ly6C^−^ CDPs showed lower ESAM and CD4 expression (Fig. 2D). In accordance with previous investigations (Onai et al., 2013), the CD115^−^Ly6C^−^ CDPs produced abundant pDCs in recipient spleen and BM, whereas CD115^+^Ly6C^−^ and CD115^+^Ly6C^+^ CDPs produced only a few and no pDCs respectively (Figs. 2A–C, S2C, and S2D). As expected, all three CDP subsets did not generate T cells, B cells, NK cells, or other myeloid cells in recipient spleen and BM (Fig. S3), reconfirming their DC-restricted differentiation capacity. Moreover, CD115^+^Ly6C^+^ CDPs also generated predominately cDC2 in nonlymphoid tissues, including the small intestine and lung (Fig. S4). Altogether, these data demonstrated that CD115^+^Ly6C^+^ CDPs represent a cDC2-committed progenitor subset within CDPs.

**Figure 2:**
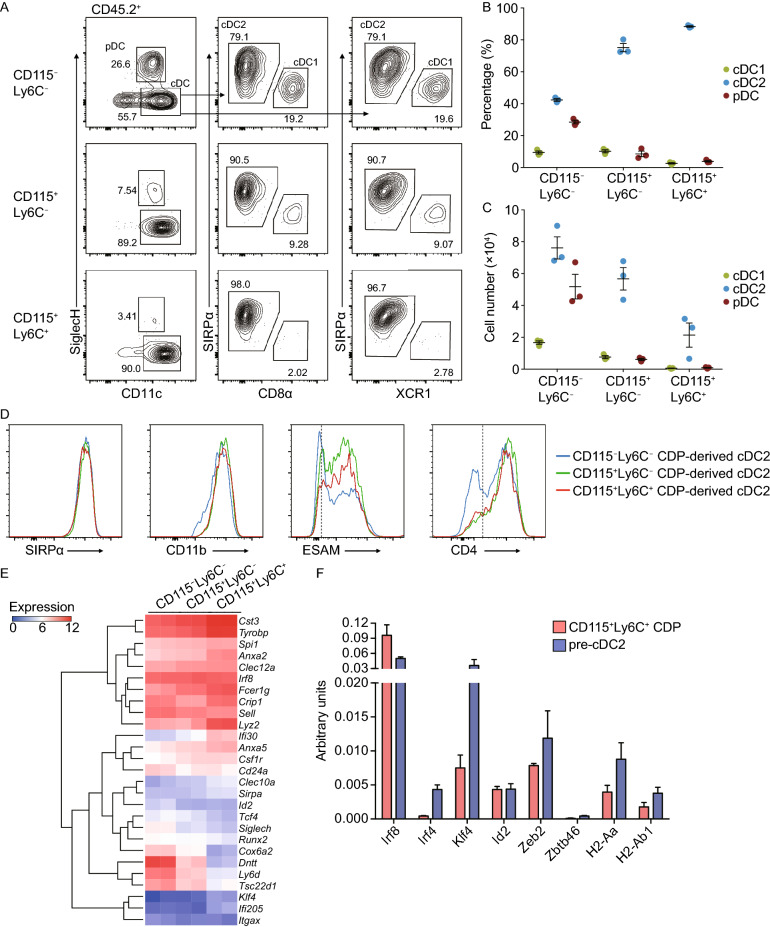
***In vivo ***differentiation potential and characterization of cDC2-primed CD115^**+**^**Ly6C**^**+**^** CDPs**. (A–C) Purified CD45.2^+^ CD115^−^Ly6C^−^, CD115^+^Ly6C^−^, and CD115^+^Ly6C^+^ CDPs (2 × 10^4^) were transplanted with CD45.1^+^ BM cells (2 × 10^5^) into irradiated CD45.1^+^ recipient mice via intravenous injection. (A) Representative flow-cytometric profiles, (B) percentages, and (C) cell numbers of splenic CD45.2^+^ DC subsets 10 days post-transplantation. (D) Expression of ESAM and CD4 on gated donor-derived CD11c^+^SIRPα^+^CD11b^+^ cDC2 cells. Dashed lines indicate positive staining threshold. (E) Log_2_-transformed (FPKM + 1) expression values of selected genes encoding products associated with DC development, assessed in CD115^−^Ly6C^−^, CD115^+^Ly6C^−^, and CD115^+^Ly6C^+^ CDPs (*n* = 2). Left margin, hierarchical clustering. (F) Expression of selected genes on purified CD115^+^Ly6C^+^ CDPs and pre-cDC2 were measured by qRT-PCR and represented by arbitrary units relative to *Actb* (*n* = 3~5). Data in (A–D) are representative of three independent experiments (*n* = 3). Data in (B, C, and F) are represented as mean ± SEM

In order to determine the differences among the three CDP subpopulations at molecular level, we compared the transcriptional signatures by RNA sequencing analysis (Fig. S5). CD115^+^Ly6C^+^ CDPs expressed higher levels of maturation-associated genes, including *Cst3*, *Fcer1g*, *Crip1*, *Ifi30*, *Anxa2*, and *Anxa5* (Schlitzer et al., 2015), consistent with their more differentiated features (Fig. 2E). Meanwhile, cDC2 signature genes, including *Sirpa*, *Clec10a*, *Tyrobp*, *Fcer1g*, *Lyz2*, *Csf1r*, and *Klf4* (Schlitzer et al., 2015), were enriched in CD115^+^Ly6C^+^ CDPs (Fig. 2E). In contrast, CD115^+^Ly6C^+^ CDPs expressed low levels of cDC1-associated *Id2* transcript and minimal levels of pDC-associated transcripts *Siglech*, *Ly6d*, *Tcf4*, and *Tsc22d1* (Schlitzer et al., 2015; Dress et al., 2019; Nutt and Chopin, 2020), further validating their cDC2-restricted differentiation potential (Fig. 2E). In addition, all three CDP subsets did not express *Itgax* (encoding CD11c), a marker expressed by pre-DCs and mature DCs, indicating that they were at a developmental stage earlier than pre-DCs (Fig. 2E). Furthermore, in terms of expression of key transcription factors involved in DC development, in comparison with pre-cDC2, CD115^+^Ly6C^+^ CDPs expressed higher levels of *Irf8* than that of pre-cDC2, but similar to that of the other two CDP subsets (Fig. 2E and 2F). Whereas the expression levels of genes associated with cDC2 differentiation *Irf4*, *Klf4*, and *Zeb2* were upregulated in pre-cDC2, confirming that pre-cDC2 were at a later stage than CD115^+^Ly6C^+^ CDPs during cDC2 differentiation. As expected, both CD115^+^Ly6C^+^ CDPs and pre-cDC2 expressed low levels of *Id2,* a cDC1 associated gene (Fig. 2F). Thus, these results demonstrated that CD115^+^Ly6C^+^ CDPs were distinct from the previously defined CD11c^+^ pre-cDC2 (Schlitzer et al., 2015).

Taken together, we identified CD115^+^Ly6C^+^ CDPs as a novel cDC2-committed progenitor subset within CDP population, and demonstrated that the commitment to cDC2 lineage occurred at the CDP stage earlier than pre-DC stage. Our findings provide novel insights into the lineage commitment of cDC2, and the CD115^+^Ly6C^+^ CDPs may serve as a potential target for modulating cDC2 differentiation and function, which will facilitate further explorations of cDC2-mediated immune modulations and therapies.

## Supplementary Information

Below is the link to the electronic supplementary material.

Supplementary file1 (DOCX 29110 kb)
