# Supplementary material for: Identification of a novel cDC2-committed progenitor within mouse common dendritic cell progenitor population
Source: Protein Cell. 2022 Jan 3;13(4):302–7. doi: 10.1007/s13238-021-00902-2 (PMC8934366; doi:10.1007/s13238-021-00902-2)
Supplement: Supplementary file 1 — Supplementary file1 (DOCX 29110 kb) [file 13238_2021_902_MOESM1_ESM.docx]

**Supplementary Information**

**Identification of a novel cDC2-committed progenitors within mouse common dendritic cell progenitor population**

Yujie Tian^1,2,3^, Xueheng Guo^1,3,4^, Tao Wu^1,3^, Kuangyu Fei^1^, Li Wu^1,3*^

^1^Institute for Immunology, Tsinghua-Peking Center for Life Sciences, School of Medicine, Tsinghua University, Beijing 100084, China

^2^Joint Graduate Program of Peking-Tsinghua-National Institute of Biological Sciences, School of Life Sciences, Tsinghua University, Beijing 100084, China

^3^Beijing Key Laboratory for Immunological Research on Chronic Diseases, Beijing 100084, China

^4^Current address: National Education Examinations Authority, Beijing 100084, China

*Correspondence: wuli@tsinghua.edu.cn

**Materials and Methods**

**Mice**

All mice used in this study were on a C57BL/6 background. All wildtype CD45.1^+^ and CD45.2^+^ mice were bred and maintained in a specific pathogen-free animal facility at Tsinghua University. The mice were maintained on a 12/12-hour light/dark cycle, 22~26 °C with sterile pellet food and water ad libitum. All animal procedures were under the approval of the Institutional Animal Care and Use Committee at Tsinghua University.

**Antibodies and flow cytometry**

Cell suspensions were incubated with polyclonal rat immunoglobulin and then labeled with fluorochrome- or biotin-conjugated monoclonal antibodies listed in the Supplementary Table 1. Cells were analyzed on LSR II (Becton Dickinson), and data were analyzed and displayed with FlowJo X software (TreeStar). Fluorescence-activated cell sorting was performed on FACSAria III (Becton Dickinson), and the purities of sorted cells were over 95%.

**Isolation of common dendritic cell progenitors (CDPs) and pre-cDC2**

Mouse bone marrow (BM) cells were collected from femur and tibia of 6~8 weeks old CD45.2^+^ wildtype mice. Erythrocytes were removed by Red Cell Removal Buffer (RCRB) (0.168 M NH_4_Cl). For CDP cell sorting, cells with a density of less than 1.086 g/cm^3^ were isolated by gradient centrifugation, followed by antibody-mediated magnetic depletion of lineage marker (CD2, CD3, CD8α, CD19, CD45R, CD11b, Ly6G, and TER119) -positive cells. The lineage marker-negative (Lin^−^) cells were stained with fluorochrome-conjugated monoclonal antibodies against Flt3, c-Kit, IL-7R, CD11c, CD115, and Ly6C. CDPs were Lin^–^c-Kit^int^Flt3^+^CD11c^–^IL-7R^–^ cells; CD115^–^Ly6C^–^, CD115^+^Ly6C^–^, and CD115^+^Ly6C^+^ CDP subsets were sorted respectively. To purify pre-cDC2, BM cells were stained by fluorochrome-conjugated monoclonal antibodies against lineage markers, MHC-II, SIRPα, Flt3, CD11c, SiglecH, and Ly6C. Lin^−^CD11c^+^MHC-II^−^CD135^+^SIRPα^−^SiglecH^−^Ly6C^+^ pre-cDC2 were sorted based on the gating strategy as previously described (Schlitzer et al. 2015).

***In vitro* DC differentiation**

Purified CD45.2^+^ CDPs (5×10^3^ or 1×10^4^) were co-cultured with CD45.1^+^ total BM cells (1.5×10^5^, erythrocytes depleted by RCRB) in RPMI 1640 medium supplemented with penicillin-streptomycin (Gibco), 10% FBS (ExCell Bio), β-mercaptoethanol (Sigma-Aldrich), and 100 ng/mL Flt3L (PeproTech) in 96-well plate as previously described (Naik et al. 2005). The progenies derived from CD45.2^+^ CDPs were analyzed by flow cytometry 3 or 6 days post culture.

**BM transplantation**

For BM transplantation, CD45.1^+^ recipient mice were lethally irradiated with two doses of 5 Gy, each approximately 2 hours apart. Donor CD45.2^+^ and CD45.1^+^ mice were sacrificed and BM cells were harvested from both femur and tibia under sterile conditions. Purified CD45.2^+^ CDPs (2×10^4^) were injected intravenously with 2×10^5^ unfractionated CD45.1^+^ BM cells (erythrocytes removed) into CD45.1^+^ recipient mice via tail vein. DC progenies were assessed 5, 10 or 15 days after BM transplantation by flow cytometry.

**Splenic and BM DC analysis**

During indicated time points post BM transfer, spleens were digested with Collagenase III (1 mg/mL, Worthington) and DNase I (0.1 mg/mL, Roche), then enriched for DCs by density separation and immunomagnetic bead depletion as previously described (Naik et al. 2006). Cells were stained with fluorochrome-conjugated monoclonal antibodies against CD45.1, CD45.2, CD11c, SiglecH, CD8α, SIRPα, and XCR1. BM cells were extracted from the femur and tibia. Erythrocytes were removed by RCRB, and debris was removed by filtration through a 70 μm strainer. BM cells were stained with fluorochrome-conjugated monoclonal antibodies against CD45.1, CD45.2, CD19, CD11b, CD11c, and SiglecH.

**Small intestine and lung DC analysis**

Lamina propria cells from recipient small intestines were isolated as previously described (Zhou et al. 2015) and then stained with fluorochrome-conjugated monoclonal antibodies against CD45.1, CD45.2, CD11c, MHC-II, CD64, CD103, and CD11b. Lungs collected from recipient mice were digested by Collagenase 1A (0.5 mg/mL, Sigma) and DNase I (0.05 mg/mL, Roche) at 37°C with several interval vortexes. Cell suspensions were collected and erythrocytes were removed by RCRB. Lung cells were stained with fluorochrome-conjugated monoclonal antibodies against CD45.1, CD45.2, CD11c, MHC-II, F4/80, CD103, and CD11b.

**Bulk RNA sequencing and data analysis**

Purified mRNA was fragmented by adding an appropriate amount of the interrupting reagent under high-temperature conditions, and the first and second strand cDNA was synthesized using the fragmented mRNA as a template. The fragment size was then selected and amplified by PCR, then the constructed library was qualified with Agilent 2100 Bioanalyzer and ABI StepOne Plus Real-Time PCR System and conducted sequencing. The raw data obtained was filtered to get clean reads. After filtering the raw data, the clean reads were aligned to the reference genome mm10 (NCBI assembly of the mouse genome) by using Bowtie2 (Langmead and Salzberg 2012), and the gene expression levels of each sample were calculated by using RSEM (Li and Dewey 2011) and normalized by calculation of the FPKM (Fragments Per Kilobase of transcript per Million fragments mapped) value. The Principal Components Analysis (PCA) was performed using the princomp function, differential expression was analyzed by DEGseq package (Wang et al. 2009) and the graphics were drawn using the R software. Heatmaps and associated hierarchical clustering were generated using Morpheus software (Broad Institute).

**Data availability**

All RNA-sequencing data have been deposited in the Gene Expression Omnibus public database under accession number GSE152350.

**Quantitative PCR**

RNA of purified progenitors was extracted by TRIzol Reagent (Invitrogen), and cDNA was generated by using PrimeScript™ RT Master Mix (Takara). A PowerUp™ SYBR™ Green Master Mix (Applied Biosystems) was used, and samples were run on an Applied Biosystems HT7900 qPCR machine. Primers were listed in Supplementary Table 2.

**Supplementary Figures**

**
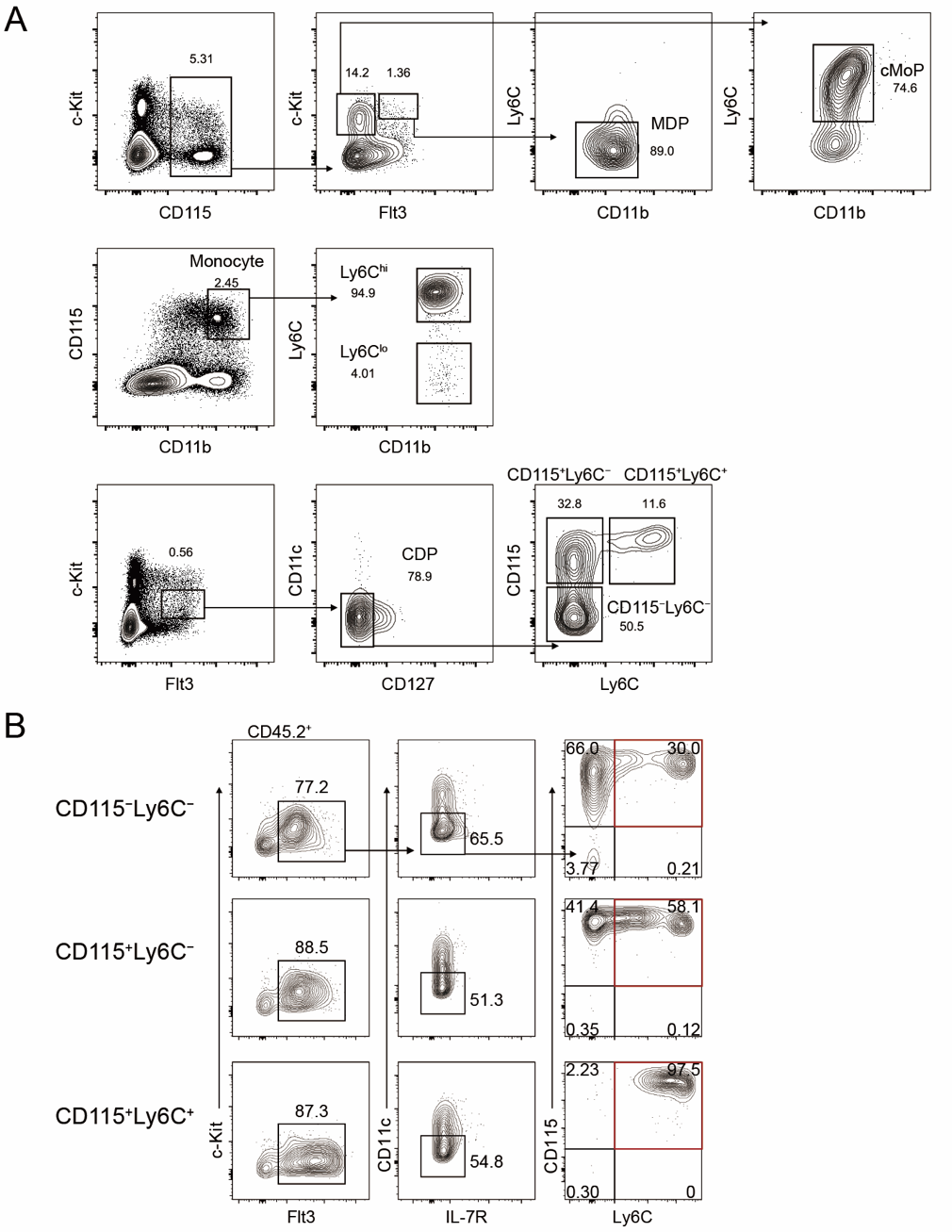
**

**Figure S1. Relationships amongst** **CD115^−^Ly6C^−^, CD115^+^Ly6C^−^, and CD115^+^Ly6C^+^ CDPs.** (A) Gating strategies used to identify MDPs, cMoPs, Ly6C^hi^ and Ly6C^lo^ monocytes, and three indicated CDP subsets in BM. (B) Purified CD45.2^+^ CD115^–^Ly6C^–^, CD115^+^Ly6C^–^, and CD115^+^Ly6C^+^ CDPs were co-cultured with CD45.1^+^ total BM cells in the medium containing Flt3L (100 ng/mL), respectively. Representative flow-cytometric profiles of CD45.2^+^ CDPs derived from each indicated CDP subset at day 1. Data are representative of three independent experiments.


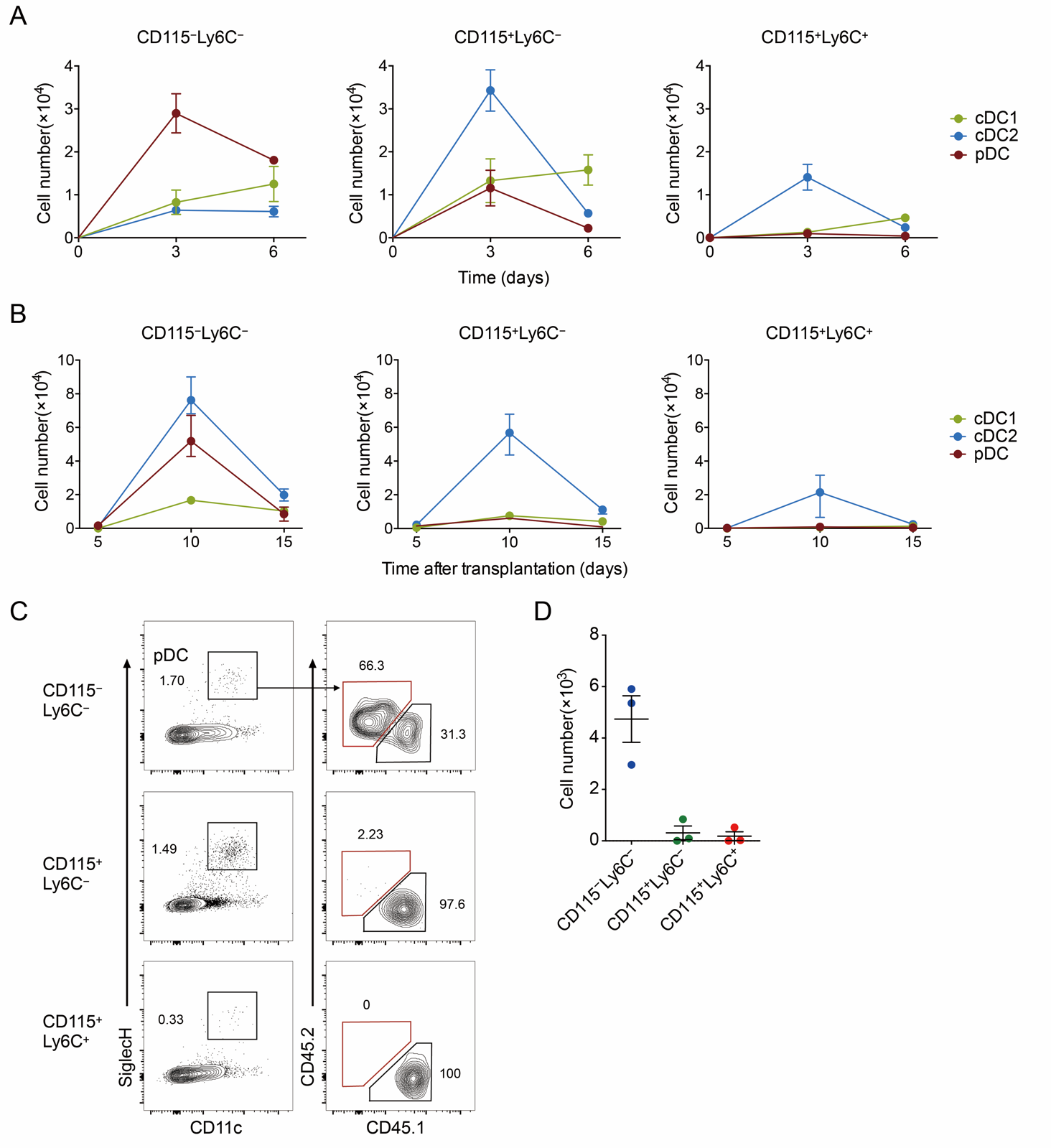


**Figure S2. DC progenies of** **CD115^−^Ly6C^−^, CD115^+^Ly6C^−^, and CD115^+^Ly6C^+^ CDPs *in vitro* and *in vivo*.** (A) Purified CD115^−^Ly6C^−^, CD115^+^Ly6C^−^, and CD115^+^Ly6C^+^ CDPs (1×10^4^) were cultured with CD45.1^+^ BM cells (1×10^5^) as feeders in the presence of Flt3L (100 ng/mL). Absolute numbers of pDC, cDC1 and cDC2 subsets on day 0, 3 and 6 of culture were calculated (*n* = 2 for each time point). (B-D) Purified CD115^−^Ly6C^−^, CD115^+^Ly6C^−^, and CD115^+^Ly6C^+^ CDPs (2×10^4^) were transplanted into irradiated CD45.1^+^ recipient mice together with CD45.1^+^ BM cells (2×10^5^) via i.v. injection, respectively. (B) Absolute numbers of splenic DC progenies from the three CDP subsets 5, 10, and 15 days post-transplantation (*n* = 2~3 for each time point). (C) Representative flow cytometric profiles and (D) absolute numbers of BM pDC progenies from the indicated CD45.2^+^ CDP subsets at day 10, gated as CD11b^−^CD19^−^CD11c^+^SiglecH^+^CD45.2^+^ (*n* = 3). Data are representative of three independent experiments. Bars represent the means with range (A, B) or the means ± SEM (D).


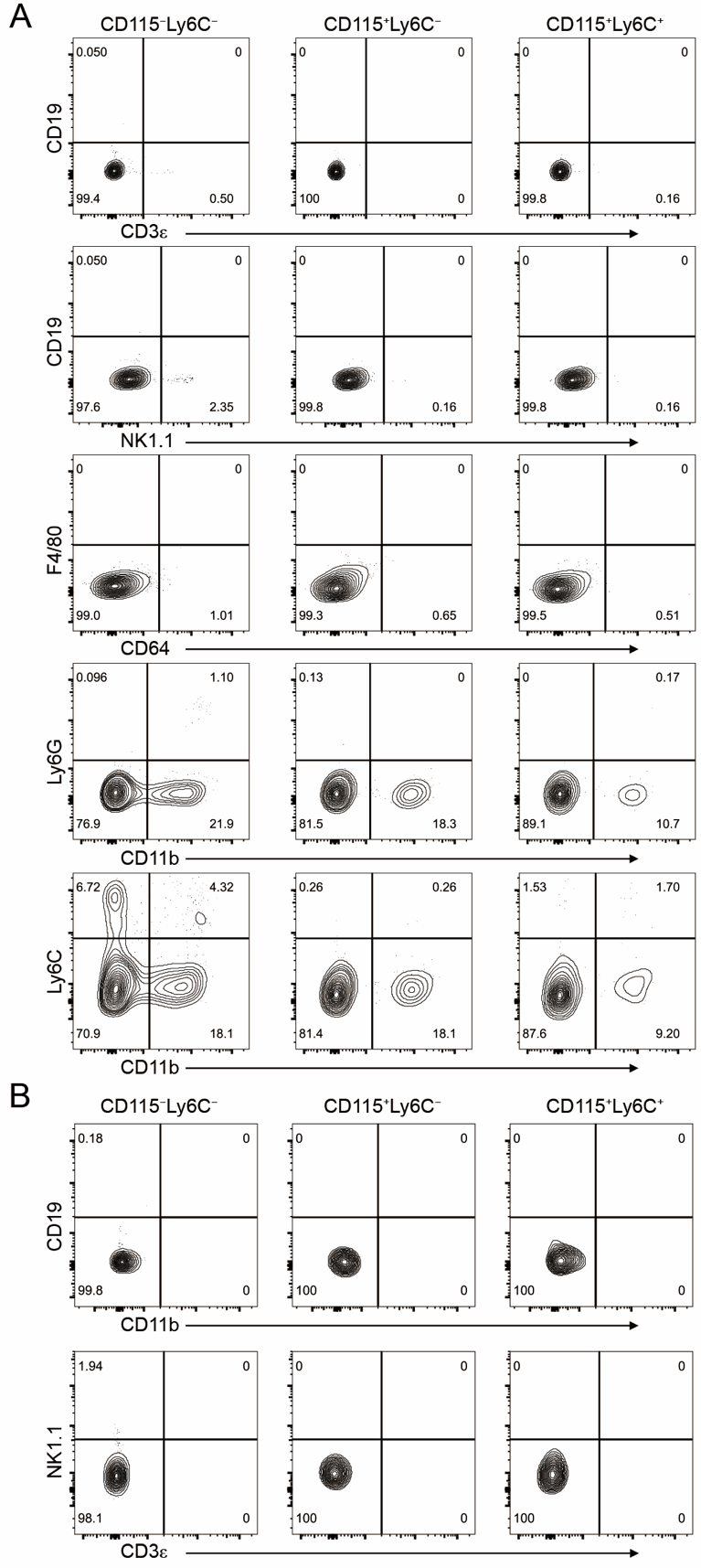


**Figure S3. All three subsets of CDPs did not generate non-DC lineage cells.** Purified CD115^−^Ly6C^−^, CD115^+^Ly6C^−^, and CD115^+^Ly6C^+^ CDPs (2×10^4^) together with CD45.1^+^ BM cells (2×10^5^) were transplanted into irradiated CD45.1^+^ recipient mice by i.v. injection, respectively (*n* = 2~3 for each group). Spleen and BM cells of the recipient mice were collected for detecting T, B, NK and myeloid progenies by flow cytometry analysis at 10 days after transplantation. (A) Flow-cytometric analysis of the CD45.2^+^ progenies in recipient spleens, by staining cells for CD3ε, CD19, NK1.1, F4/80, CD64, Ly6G, Ly6C, and CD11b. (B) Flow-cytometric analysis of the CD45.2^+^ progenies in recipient BM, by staining cells for CD19, CD11b, CD3ε, and NK1.1.


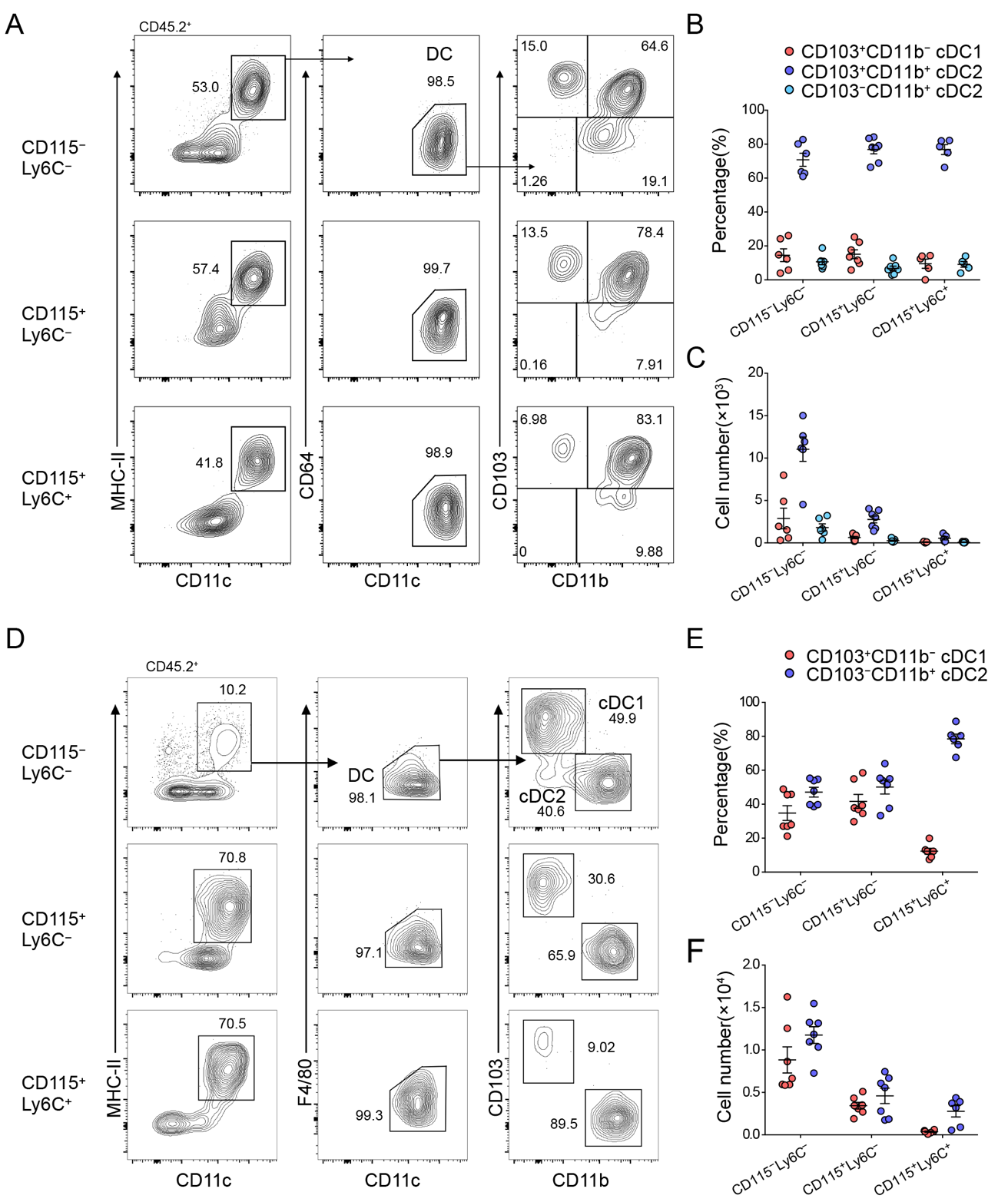


**Figure S4. CD115^+^Ly6C^+^ CDPs preferentially gave rise to cDC2 in the small intestine and lung.** Purified CD115^−^Ly6C^−^, CD115^+^Ly6C^−^, and CD115^+^Ly6C^+^ CDPs (2×10^4^) were transplanted together with CD45.1^+^ BM cells (2×10^5^) into irradiated CD45.1^+^ recipient mice via i.v. injection, respectively. Small intestine (SI) and lung samples were subjected to flow cytometry analysis for DC progenies at 10 days after transplantation. (A) Flow-cytometric profiles of the CD45.2^+^ DC subsets in recipient SI. Progenies were stained for CD11c, MHC-II, CD64, CD103, and CD11b to distinguish each cDC subset. (B) Percentages of each SI DC subset within CD45.2^+^CD11c^+^MHC-II^+^ cells and (C) cell numbers of each SI DC subset derived from the indicated CDP subsets. (D) Flow-cytometric profile of the CD45.2^+^ DC subsets in recipient lung. Progenies were stained for CD11c, MHC-II, F4/80, CD103, and CD11b to distinguish each cDC subset. (E) Percentages of each lung DC subsets within CD45.2^+^CD11c^+^MHC-II^+^ cells and (F) cell numbers of each DC subset in lung derived from the indicated CDP subsets. Data were integrated from three independent experiments (*n* = 5~8). Bars represent the means ± SEM.


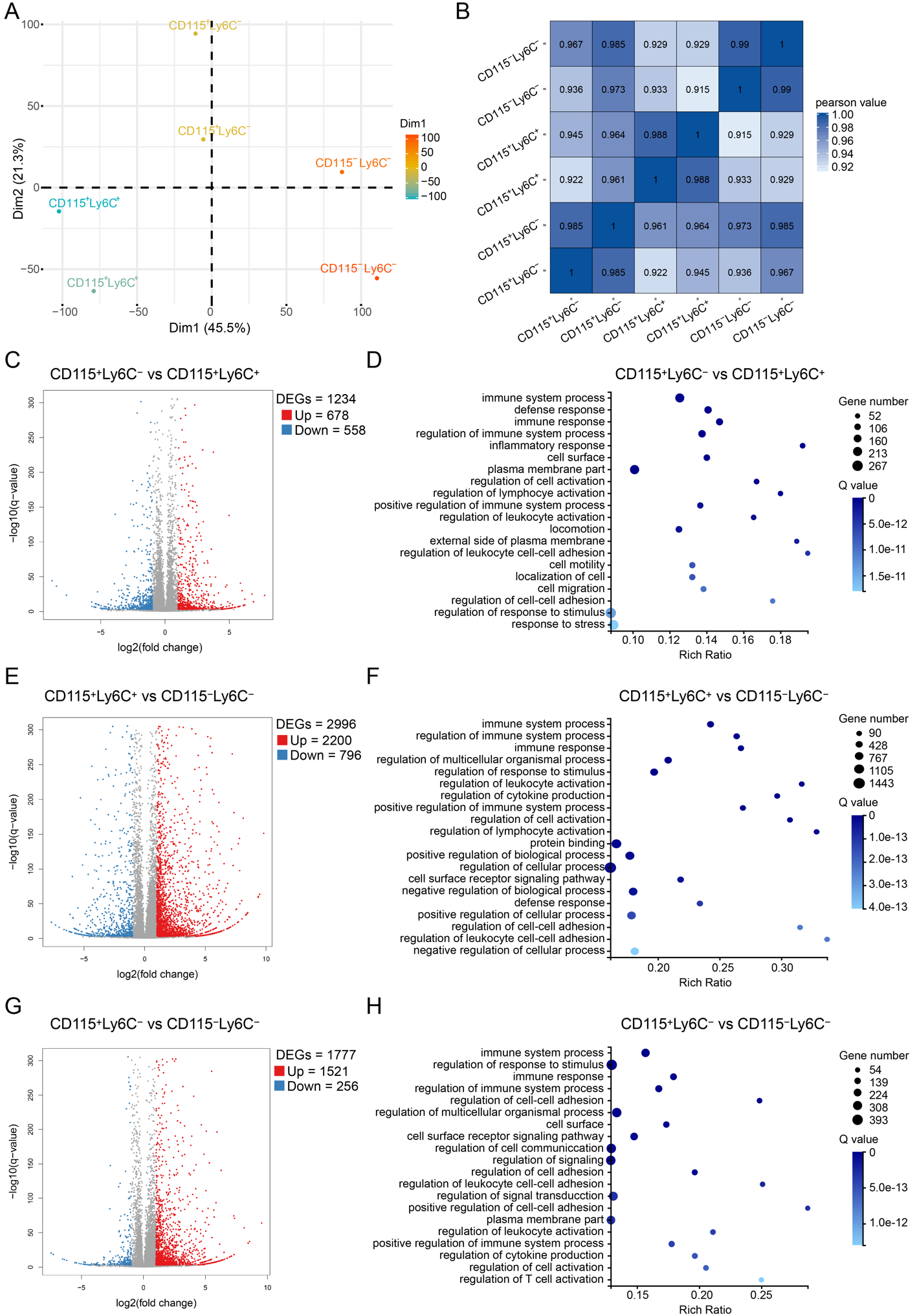


**Figure S5. CD115^−^Ly6C^−^, CD115^+^Ly6C^−^, and CD115^+^Ly6C^+^ CDP subpopulations were transcriptionally related.** Bulk RNA sequencing was performed on purified CD115^−^Ly6C^−^, CD115^+^Ly6C^−^, and CD115^+^Ly6C^+^ CDPs; *n* = 2 per cell type. (A) PCA of expression profiles on all expressed genes in three CDP subsets; dim1 and dim2 (along axes) represent the largest two principal components. (B) Correlation heatmap based on Pearson correlation coefficients calculated on all gene expressions between each pair of samples. (C-H) Pairwise comparisons of expression profiles among the three CDP subsets. (C, E, G) Volcano plots visualizing the extent of differential expressions between indicated CDP subsets. The X-axis represents the log_2_ fold change, while the Y-axis depicts significance (-log_10_-transformed *q* values). Red dots represent up-regulated differentially expressed genes (DEGs) (fold change ≥ 2 and *q*-value ≤ 0.001), blue dots represent down-regulated DEGs (fold change ≤ −2 and *q*-value ≤ 0.001), and gray dots represent no-DEGs (absolute fold change < 2 or *q*-value > 0.001). (D, F, H) The enrichment bubble charts displaying the GO enrichment results of the DEGs between indicated CDP subsets (Top 20 GO Terms with the smallest *q*-values). The X-axis represents the enrichment ratio (Rich Ratio) and the Y-axis depicts GO Term. The size of the bubble indicates the number of DEGs annotated to a GO Term. The color represents the enriched *q*-value; the darker the color, the smaller the *q*-value.

**Supplemental Table 1. Antibody list for flow cytometry**

| Antibody | Clone | Supplier | Conjugates | Catalog No. |
| --- | --- | --- | --- | --- |
| CD135 (Flt3) | A2F10 | eBioscience | PE | 12-1351-83 |
| CD103 | 2E7 | eBioscience | PE | 12-1031-83 |
| CD115 | AFS98 | eBioscience | Biotin | 13-1152-82 |
| CD117 (c-Kit) | ACK2 | BioLegend | Brilliant Violet 605 | 135121 |
| CD11b | M1/70 | BioLegend | Brilliant Violet 605 | 101237 |
| CD11c | N418 | eBioscience | FITC | 11-0114-85 |
| CD11c | N418 | eBioscience | PE-Cy7 | 25-0114-82 |
| IL-7Rα | A7R34 | BioLegend | Brilliant Violet 421 | 135024 |
| CD172α (SIRPα) | P84 | eBioscience | APC | 17-1721-82 |
| CD172α (SIRPα) | P84 | BD Biosciences | FITC | 560316 |
| CD19 | eBio1D3 | eBioscience | APC | 17-0193-82 |
| CD19 | 6D5 | BioLegend | Pacific Blue | 115523 |
| CD2 | RM2-5 | BioLegend | PerCP/Cy5.5 | 100115 |
| CD24 | M1/69 | BioLegend | Brilliant Violet 605 | 101827 |
| CD3 | 17A2 | BioLegend | PerCP/Cy5.5 | 100217 |
| CD4 | RM4-5 | BioLegend | Brilliant Violet 711 | 100550 |
| CD45.1 | A20 | eBioscience | PE-Cy7 | 25-0453-81 |
| CD45.1 | A20 | BioLegend | Brilliant Violet 711 | 110739 |
| CD45.2 | 104 | BioLegend | APC-Cy7 | 109824 |
| CD45R | RA3-6B2 | eBioscience | PE-Cy7 | 25-0452-82 |
| CD45R | RA3-6B2 | BioLegend | PerCP/Cy5.5 | 103235 |
| CD64 | X54-5/7.1 | BioLegend | Brilliant Violet 711 | 139311 |
| CD8α | 53-6.7 | BioLegend | Brilliant Violet 605 | 100744 |
| CD8α | 53-6.7 | BioLegend | PerCP/Cy5.5 | 100733 |
| ESAM | 1G8 | BioLegend | PE | 136203 |
| F4/80 | BM8 | eBioscience | APC | 17-4801-82 |
| Ly6C | HK1.4 | eBioscience | APC | 17-5932-82 |
| Ly6C | HK1.4 | eBioscience | eFluor 450 | 48-5932-82 |
| Ly6G | 1A8 | eBioscience | FITC | 11-9668-82 |
| MHC-II | M5/114.15.2 | BioLegend | Pacific Blue | 107620 |
| MHC-II | M5/114.15.2 | eBioscience | FITC | 11-5321-82 |
| SiglecH | eBio440C | eBioscience | PE | 12-0333-82 |
| SiglecH | 551 | BioLegend | APC | 129612 |
| Streptavidin |  | eBioscience | PE-Cy7 | 25-4317-82 |
| TER119 | TER-119 | BioLegend | PerCP/Cy5.5 | 116227 |
| XCR1 | ZET | BioLegend | Brilliant Violet 421 | 148216 |

**Supplemental Table 2. Primers used for qPCR**

| Gene | Forward Primer (5'-3') | Reverse Primer (5'-3') |
| --- | --- | --- |
| *Irf4* | TCCGACAGTGGTTGATCGAC | CCTCACGATTGTAGTCCTGCTT |
| *Irf8* | CGGGGCTGATCTGGGAAAAT | CACAGCGTAACCTCGTCTTC |
| *Klf4* | GTGCCCCGACTAACCGTTG | GTCGTTGAACTCCTCGGTCT |
| *Id2* | ATGAAAGCCTTCAGTCCGGTG | AGCAGACTCATCGGGTCGT |
| *Zeb2* | GGCAAGGCCTTCAAGTACAA | AAGCGTTTCTTGCAGTTTGG |
| *Zbtb46* | GACACATGCGCTCACATACTG | TGCACACGTACTTCTTGTCCT |
| *H2-Aa* | TCAGTCGCAGACGGTGTTTAT | GGGGGCTGGAATCTCAGGT |
| *H2-Ab1* | AGCCCCATCACTGTGGAGT | GATGCCGCTCAACATCTTGC |
| *Actb* | AGAGGGAAATCGTGCGTGAC | CAATAGTGATGATGACCTGGCCGT |

**References**

Langmead B, Salzberg SL (2012) Fast gapped-read alignment with Bowtie 2. Nat Methods 9:357–359.

Li B, Dewey CN (2011) RSEM: Accurate transcript quantification from RNA-Seq data with or without a reference genome. BMC Bioinformatics 12:323.

Naik SH, Metcalf D, van Nieuwenhuijze A, et al (2006) Intrasplenic steady-state dendritic cell precursors that are distinct from monocytes. Nat Immunol 7:663–671.

Naik SH, Proietto AI, Wilson NS, et al (2005) Cutting Edge: Generation of Splenic CD8 + and CD8 − Dendritic Cell Equivalents in Fms-Like Tyrosine Kinase 3 Ligand Bone Marrow Cultures. J Immunol 174:6592–6597.

Schlitzer A, Sivakamasundari V, Chen J, et al (2015) Identification of cDC1- and cDC2-committed DC progenitors reveals early lineage priming at the common DC progenitor stage in the bone marrow. Nat Immunol 16:718–728.

Wang L, Feng Z, Wang X, et al (2009) DEGseq: An R package for identifying differentially expressed genes from RNA-seq data. Bioinformatics 26:136–138.

Zhou H, Xiao J, Wu N, et al (2015) MicroRNA-223 Regulates the Differentiation and Function of Intestinal Dendritic Cells and Macrophages by Targeting C/EBPβ. Cell Rep 13:1149–1160.
